# The patient experience of a postpartum readmission for hypertension: a qualitative study

**DOI:** 10.1186/s12884-024-06564-2

**Published:** 2024-05-14

**Authors:** Radhika Viswanathan, Sarah E. Little, Louise Wilkins-Haug, Ellen W. Seely, Saba H. Berhie

**Affiliations:** 1 SUNY Downstate Health Sciences University, 450 Clarkson Avenue, Brooklyn, NY 11203 USA; 2 Department of Obstetrics, Gynecology and Reproductive Science, Mount Sinai Hospital, New York, NY USA; 3 Department of Obstetrics and Gynecology, Brigham and Women’s Hospital, Boston, MA USA; 4 Department of Medicine, Brigham and Women’s Hospital, Boston, MA USA

**Keywords:** Hypertensive disorders of pregnancy, Postpartum readmission, Qualitative research, Patient experience

## Abstract

**Background:**

Hypertensive disorders of pregnancy (HDP) are the most common cause of postpartum readmission. Prior research led to clinical guidelines for postpartum management; however, the patient experience is often missing from this work. The objective of this study is to understand the perspective of patients readmitted for postpartum hypertension.

**Methods:**

This was a qualitative study with data generated through semi-structured interviews. Patients readmitted with postpartum HDP at an urban academic medical center from February to December 2022 were approached and consented for an interview. The same researcher conducted all interviews and patient recruitment continued until thematic saturation was reached (*n* = 9). Two coders coded all interviews using Nvivo software with both deductive and inductive coding processes. Discrepancies were discussed and resolved with consensus among the two coders. Themes were identified through an initial a priori template of codes which were expanded upon using grounded theory, and researchers were reflexive in their thematic generation.

**Results:**

Six themes were generated: every pregnancy is different, symptoms of preeclampsia are easily dismissed or minimized by both patient and providers, miscommunication regarding medical changes can increase the risk of readmissions, postpartum care coordination and readmission logistics at our hospital could be improved to facilitate caring for a newborn, postpartum care is often considered separately from the rest of pregnancy, and patient well-being improved when conversations acknowledged the struggles of readmission.

**Conclusions:**

This qualitative research study revealed patient-identified gaps in care that may have led to readmission for hypertensive disorders of pregnancy. The specific recommendations that emerge from these themes include addressing barriers to blood pressure management prior to discharge, improving postpartum discharge follow-up, providing newborn care coordination, and improving counseling on the risk of postpartum preeclampsia during discharge. Incorporating these patient perspectives in hospital discharge policy can be helpful in creating patient-centered systems of care and may help reduce rates of readmission.

**Supplementary Information:**

The online version contains supplementary material available at 10.1186/s12884-024-06564-2.

## Background

The fourth trimester, or the first three months postpartum, is a time fraught with challenges for the birthing person and has the potential for medical morbidity and mortality. Hypertensive disorders of pregnancy (HDP), defined as either chronic or pregnancy-associated hypertension, complicate between 5 and 13% of pregnancies [1], and are the most common cause of postpartum readmissions [2]. Further, they can cause up to 7% of maternal mortality, 70% of which occurs in the postpartum period [3]. At our facility, we have begun a follow-up program for postpartum people with hypertensive disorders of pregnancy. In this cohort of 164 postpartum people with hypertensive disorders of pregnancy who (1) delivered from 2/2023 to 6/2023 and (2) received care in our faculty MFM and Ob-Gyn practice there were 19 readmissions (11.6%). Readmission due to postpartum hypertension within 6 weeks of delivery can cost the healthcare system up to $700 million. It also can cause strain for the mother-child dyad as there may be separation and interruption of bonding in the early postpartum period [4]. Given the high prevalence of HDP among readmitted patients and burden that readmissions place on patients, families, and the health care system, it is important to understand what factors can be modified to reduce these rates. Prior research led to the creation of clinical guidelines for postpartum management [5]; however, the patient experience is often missing from this work.

Risk factors and characteristics of readmitted postpartum patients have been delineated in prior studies [6, 7]. Readmitted patients are more likely to be publicly insured, Black, have maternal comorbidities such as psychiatric disease, seizure disorder, or hypertension, have had a c-section, or complications such as infections and thrombotic events. Patients who were readmitted for a postpartum HDP were more likely to have had a long duration of labor and to have been started on antihypertensive during their pregnancy compared to patients who were not readmitted [7].

Qualitative investigations have been conducted during the postpartum period. A focus group study that specifically concentrated on provider-patient communication about preeclampsia found that patients believed communication could be improved with flyers, access to online communities about preeclampsia, incorporating their families in conversations about managing blood pressure, and better education of primary care providers about preeclampsia [8]. Another study used semi-structured interviews to examine the barriers and facilitators to postpartum care among patients diagnosed with gestational diabetes. General barriers to care that emerged were worries about their baby’s health issues, fear of a diabetes diagnosis, misunderstanding of the disease, and mental health concerns [9]. We hypothesize that similar themes could also play a role when it comes to lack of follow-up for patients with postpartum hypertension, leading to increased unnecessary readmissions.

It is recommended that women discharged after delivery, especially women with risk factors for HDP, be counseled on the risk of postpartum hypertension and demonstrate understanding of the risks of postpartum hypertension to reduce readmission rates [2]. However, to date, no qualitative studies looking at the relationship between postpartum readmission for hypertension and patient understanding has been conducted. The patient perspective is a necessary piece of the story to clarify the gaps in communication between providers and patients and inform interventions targeting postpartum readmission rates. Thus, our goal was to understand the patient perspective on their postpartum readmission.

## Methods

Women aged 18 years of older and a HDP diagnosis with a postpartum readmission secondary to HDP within six weeks of delivery at an urban academic medical center were approached and consented for an interview between February and December 2022. Individuals were selected by the study team through examination of the daily list of postpartum patients admitted to the hospital to identify any readmissions for hypertension. Study staff approached the patient during their readmission and verbal agreement was approved as consent by the IRB. All English-speaking readmitted patients were eligible. Patients who are readmitted for postpartum hypertension were the study population given the goals of the study. Participants were renumerated with a gift card of $25 for participating. The participants were interviewed either in person during their readmission or over a phone call within a week of their discharge after readmission.

A semi-structured interview method was chosen to allow the most flexibility in the data collection process and allow participant responses to guide the study questions and analysis. An interview guide, included as supplementary material, was developed specifically to foster discussion around the delivery experience, readmission process, factors that could have prevented the readmission, challenges around childcare, challenges in blood pressure management, and patient understanding of the risks of postpartum preeclampsia.

Each interview was conducted, recorded, and transcribed by the same author (RV), and participant recruitment continued until thematic saturation was achieved (*n* = 9). Each interview lasted approximately 30 min. Participants were notified that their recordings would be deidentified and were informed that they could end the interview at any time. The study was approved by the Brigham and Women’s Hospital Institutional Review Board.

### Data analysis

Two coders (RV and SHB) independently read and re-read the interviews, using Nvivo software to code the interviews through a hybrid approach of deductive and inductive coding processes which has been validated in prior literature [10]. An initial codebook was created through a directed content analysis deductive process. During the coding of transcripts, inductive codes were created using the grounded theory approach to better qualify the patient’s lived experiences. The final codes were created based on repeated words and opinions, similarities in responses to specific targeted questions, and general thought patterns. Through grounded theory, the same two coders created categories from these codes and identified themes that emerge. Discrepancies were discussed and resolved with consensus. Themes were identified through grounded theory, and researchers were reflexive in their thematic generation.

## Results

Nine patients were consented and agreed to be interviewed for the study. Four of the patients were interviewed during their readmission in the hospital and 5 were interviewed at home after they had been discharged, within a week of their discharge. Patient demographics are included in Table 1 and information regarding postpartum follow-up is included in Table 2.

**Table 1:** Patient characteristics

Patient number	Age	Race/ethnicity	Postpartum day (upon readmission)	History of hypertension prior to this pregnancy	Interview method
1	32	White, non-Hispanic	3	No	In person
2	36	Black, non-Hispanic	4	Yes	In person
3	35	White, non-Hispanic	7	Yes	In person
4	39	White, Hispanic	4	Yes	Telephone
5	41	White, non-Hispanic	3	No	Telephone
6	37	White, non-Hispanic	5	Yes	In person
7	35	Black, non-Hispanic	7	No	Telephone
8	31	White, non-Hispanic	8	No	Telephone
9	29	White, non-Hispanic	4	Yes	Telephone

**Table 2:** Postpartum follow-up

Patient number	Follow-up scheduled within a week of delivery?	Patient attended 1-week postpartum visit?
1	No	N/A - readmitted prior to scheduled visit
2	Yes	Yes
3	Yes	Yes
4	Yes	No
5	Yes	Yes
6	Yes	Yes
7	No	N/A - readmitted prior to scheduled visit
8	No	N/A - readmitted prior to scheduled visit
9	No	N/A - readmitted prior to scheduled visit

Themes are included below, according to the major categories that were explored through the semi-structured interviews. A summary of the themes is included in Table 3.

### Every pregnancy is different

The range of responses from participants who had a previous pregnancy prior to this delivery and readmission was varied based on lived experience. Some patients had never been diagnosed with an HDP: “*I’ve never had high blood pressure, so I’ve never had any symptoms of high blood pressure either. I was fine with blood pressures during my whole pregnancy.”* Other participants had had a prior diagnosis of preeclampsia in a previous pregnancy and could recognize symptoms:

I feel like I was in a maybe unique situation because I had been monitored for this for so long that I knew exactly what to look for, you know: headaches, blurry vision, right epigastric pain, increasing blood pressure… so I kind of knew the drill by that point.

Still others had a prior diagnosis of preeclampsia in a prior pregnancy but had experienced different symptoms: *“When I had the situation from the first pregnancy, I didn’t have any of these symptoms…”*.

### Symptoms of preeclampsia are easily dismissed or minimized by both patients and providers

Several patients noted that due to the nonspecific and varied nature of preeclampsia symptoms, either they or their healthcare providers dismissed the symptoms as less serious than they were. One patient reported mistaking right upper quadrant abdominal pain:

I asked [the urgent care], ‘Do you think it could be just like gas and bloating,’ and they said, ‘probably’, because my vitals were fine, everything else wasn’t indicating anything serious. So, I just took over-the-counter gas-ex medication to see if it helped, but it wasn’t improving.

Another patient reported that she had expected to feel bad simply because she had just delivered a baby: *“My family saw me and they were just like, ‘oh wow you look terrible,’ and I was like ‘well I just had a kid.’”* Yet another patient attributed her shortness of breath to other etiologies: *“My toddler had a bit of a cold, and I started wheezing at night, maybe two nights or three nights later. And I just figured it was a flareup of my asthma.”*

### Miscommunication regarding medical care can contribute to the risk of HDP readmissions

Several patients reported instances of miscommunication between them and their medical providers, leading to misunderstandings around their risk of developing a postpartum HDP, as well as misunderstandings around the severity of such a diagnosis. For example, several patients were unaware that a HDP was a possibility after delivery:

Because I knew that preeclampsia can happen obviously while you’re pregnant or can happen like right after you deliver within the first 24–48 h. I had no clue that it could happen a week later. That was the last thing that was on my mind.

A major contributor to potential miscommunication is the amount of information patients are given during their labor and delivery admission. Patients acknowledged that they were overwhelmed after their delivery, so they may not have processed everything that was communicated during their discharge.

After I delivered, everything was a bit of a blur… I think maybe just because of the moment I was in, I may have been more focused on all of the information about the baby… I do remember them saying something about my blood pressure and I will be honest in that moment it did not really register with me.

Another patient described leaving against medical advice after her delivery because she felt that there were daily changes to her medical care that she did not feel that were being communicated well.

Why would you give me a new pill and not say anything? [Why would you] say that I could leave at 10am but now you’re saying basically I have to stay another day? So that really bothered me. I was at that point where I just wanted to go. I didn’t want to stay because it was just, something that you should have said before.

### Postpartum care coordination and readmission logistics at our hospital could be improved to facilitate caring for a newborn

Three patients reported that it was difficult to consider on their own health needs while they were focusing on their newborn’s care: “*I was focused on… actively making appointments for the baby and I was not thinking about appointments for me beyond that one that was going to happen at 6 weeks.”* Two patients noted that a visiting nurse system was very helpful to catch symptoms that they would have otherwise dismissed. One patient suggested that having a virtual visit a few days after delivery would have been useful for her to bring up questions to her providers.

With regards to their readmission, several patients noted that a major stressor around the readmission experience was the need to care for their newborn. Our institution’s policy for postpartum magnesium sulfate administration is at the admitting physician’s discretion. Generally, a patient readmitted with neurological symptoms in the setting of hypertension will receive 12 h of magnesium sulfate. When a postpartum person is readmitted on magnesium their newborn is only allowed to be in the room with them if another adult caregiver is present per hospital policy. Patients expressed fear and nervousness around this stressor: *“I was nervous and scared because I just didn’t want to leave my husband by himself. Being hormonal and having a newborn at home, that was a big part of it.”* Some patients indicated the unknown of childcare logistics being their utmost priority even as they were being admitted. If the patient’s family members were visiting, there were further challenges around how to care for the baby in the hospital: *“So, [my husband] couldn’t order food, there was no mechanism for him to literally go and get food that didn’t involve toting the baby around the hospital.”*

### Postpartum care is often considered separately from the rest of pregnancy

Interviews revealed several examples of how patient care in the postpartum period was kept separate from other antepartum and labor and delivery care. One patient reported that the process of being admitted as a postpartum patient required waiting in the adult emergency room rather than being taken to the labor and delivery triage, which is where she which is where she felt she received more specialized postpartum care. Several patients reported feeling that after the delivery, they felt like they were no longer at risk for complications:

I don’t know if I sort of thought that I was out of the woods on the preeclampsia, just because, ‘Hey, I delivered, the kid’s here, we made it.’ So, this was a little bit of a surprise and a shock to me.

### Patient well-being improved when conversations acknowledged the struggles of a readmission

Patients explained that their readmission experience was most supported by conversations that suggested providers could empathize with the patients’ situations:

The doctor asked me, ‘What is your biggest worry or concern right now?’ Because they read my face, they knew I was concerned… that was helpful to have her here, to have somebody to care enough to even ask me.

Many patients who were interviewed felt ultimately grateful for their symptoms being caught and treated in a timely manner:

Because it could have been a really bad outcome. I read more about it after being admitted and it’s just scary. I think everyone did the best that they could with our care, and this was just an unexpected bump in the road.

### Exploration of perspectives by place of interview (inpatient versus outpatient)

We did not find differences in perspectives between postpartum people interviewed in the inpatient setting or outpatient setting. We anticipated that stressors may differ between groups, namely that childcare would be more salient for the inpatient cohort, however, both groups brought up childcare concerns without prompting.

- Quote from inpatient interviewee: *“When you have a family at home that you have to care for, a newborn that you have to feed, and you’re being admitted into the hospital, it’s very very stressful. You’re trying to figure out who’s going to care for these kids.”*
- Quote from outpatient interviewee: *“I think I was just really, I was nervous and scared because I just didn’t want to leave my husband by myself. Being hormonal and having a newborn at home, that was a big part of it.”*

**Table 3:** Themes Generated from Patient Interviews

Theme	Representative Quote
Every pregnancy is different.	*“When I had the situation from the first pregnancy, I didn’t have any of these symptoms. The only symptom I had was swelling. I didn’t have any headache, no blurred vision, no abdominal pain, no anything, so I just thought it was all just normal.”*
Symptoms of preeclampsia symptoms are easily dismissed or minimized by both patients and providers.	*“I asked them, ‘Do you think it could be just like gas and bloating,’ and they said, ‘probably,’ because my vitals were fine, everything else wasn’t indicating anything serious. So, I just took over-the-counter gas-ex medication to see if it helped, but it wasn’t improving.”*
Miscommunication regarding medical care can contribute to the risk of HDP readmissions.	*“After I delivered, everything was a bit of a blur… I do remember them saying something about my blood pressure and I will be honest in that moment it did not really register with me.”*
Postpartum care coordination and readmission logistics at our hospital could be improved to facilitate caring for a newborn.	*“So, [my husband] couldn’t order food, there was no mechanism for him to literally go and get food that didn’t involve toting the baby around the hospital.”*
Postpartum care is often considered separately from the rest of pregnancy.	*“I don’t know if I sort of thought that I was out of the woods on the preeclampsia, just because, ‘Hey, I delivered, the kid’s here, we made it.’ So, this was a little bit of a surprise and a shock to me.”*
Patient well-being improved when conversations acknowledged the struggles of readmission.	*“The doctor asked me, ‘What is your most worry or concern right now?’ Because they read my face, they knew I was concerned.”*

## Discussion

This study elucidates several key patient-centered factors that could be related to a readmission for postpartum HDP. One important factor is misunderstanding of the symptoms and risks of postpartum HDP. Patients reported that symptoms of postpartum preeclampsia were non-specific, and either they or medical providers would minimize their symptoms. Additionally, several patients expressed that they were very overwhelmed after their deliveries and may not have processed warning signs and counseling during their initial discharge. Because the labor and delivery admission can be a multi-layered and challenging experience, it may not be sufficient to solely inform the patient about the risks surrounding the postpartum period during their labor and delivery discharge. A tool that includes formal and longitudinal patient education has been shown to improve blood pressure control in the postpartum period [11]. Similarly, introducing anticipatory guidance in the antenatal period, followed with formal patient education during the initial postpartum visit (either virtually or in person) would be a better environment to provide in-depth information about the risks as well as the signs and symptoms of a postpartum HDP. Patients found the use of visiting nurses and telemedicine helpful, so these resources should continue to be incorporated in postpartum follow-up.

All patients in our study were within 10 days postpartum when they were readmitted. This suggests that an early postpartum visit could be helpful in catching most high-risk patients. ACOG has proposed a paradigm shift in postpartum management to introduce a blood pressure check within the first 3–10 days postpartum, as well as an initial contact with all women within the first three weeks postpartum [5]. These guidelines would likely allow providers to intervene in care early before a patient requires a readmission for postpartum HDP. Incorporating telemedicine could be a way to improve access to postpartum care in a timely manner.

Regarding the postpartum experience, patients noted that their primary concern was around the well-being and logistics around care and feeding of their newborn. At our institution, when a postpartum patient is readmitted, the baby can only stay with them if another adult is available to be the primary caretaker. During a maternal readmission the baby also does not have access to the newborn nursery and nursing support systems. Creating a hospital environment that promotes rooming in with the baby during a readmission could improve this experience if implemented appropriately [12]. At the very least, patients noted that simply acknowledging the stressors surrounding being admitted while being a new parent helped them feel more supported during the readmission.

Other ways to ease the burden of care on new mothers would be better integrating postpartum and newborn care. One study found that using well-child visits to provide inter-conception education, including screening for mental health issues, tobacco use, and contraception use, successfully addressed certain behavior risks in the mother [13]. The American Academy of Pediatrics also recommends maternal mental health screening during newborn pediatric visits [14]. Similar novel models of healthcare delivery that combine postpartum and pediatric care could be used to streamline education on postpartum hypertension.

Results of this study align well with other research, which have shown that worries about the newborn’s health issues often supersede the patient’s own health concerns, that major barriers to seeking care include a lack of understanding of the risks of preeclampsia, and that patients are often only aware of preeclampsia if they had been diagnosed in a prior pregnancy [15, 16]. Finally, qualitative literature on the postpartum period overall suggests that patients feel that their needs are often overlooked during this time [17]. The conversations in our study suggested reasons why patients may minimize or misunderstand the risks that may be present in the postpartum period.

### Limitations

An important limitation of this study is that, due to the selection criteria, we were only able to recruit patients who presented for readmission. Therefore, we are not able to include the perspective of patients who had a postpartum HDP but did not present to the hospital, due to other barriers that prevented them from accessing care or those who were managed as outpatients. For example, all the patients that we spoke to emphasize the importance of having family support to care for their newborn and/or other children during their readmission. New mothers without this family support may have additional barriers to seeking care even if they did experience symptoms of an HDP.

Other limitations include the patient demographics; the patients who were recruited were mostly Caucasian, all spoke English, and were all admitted to the same urban academic hospital. As a result, this study directly reflects the experiences of these patients and is most useful for quality improvement endeavors at this or similar academic centers. Many of the challenges that these patients expressed were specific to hospital policy; for example, barriers to rooming in while on magnesium may not be present at other hospitals. Additionally, we were not able to interview every patient who was eligible during the study period as some eligible patients were discharged before they could be approached and consented. Finally, patients were interviewed via a mix of in person and telephone calls at their convenience. For those who were interviewed in person, physical cues may have been conveyed between the participant and the interviewer that could have affected participant responses in the conversation. Similarly, these physical cues may have been missed over a telephone call, which could have changed the trajectory of the conversation. Additionally, patients in the inpatient setting during their readmission may have a different perspective on their readmission than those who have returned home after their readmission; however, all patients were interviewed within a week of their readmission. One theme that we considered could be different between the two groups was with regards to childcare, but patients interviewed both in the inpatient and outpatient setting brought up childcare concerns without prompting.

## Conclusion

The findings of this study can lead to actionable recommendations that could be implemented (1) during discharge planning and in the immediate postpartum period to potentially reduce rates of readmission and (2) during a readmission to improve the patient experience. Patients could be given clearer anticipatory guidance around the risks of postpartum HDP towards the end of their pregnancy rather than only upon discharge; a standardized postpartum assessment within 1 week of delivery should be implemented to assess signs and symptoms of an HDP. For those patients who need a readmission, hospitals can implement policies to allow babies to room in with the mother and emphasize an empathetic approach when providers communicate with patients.

The themes that emerged from the study suggest that patients in the postpartum period have unique barriers to understanding their medical risks and accessing postpartum care. Therefore, additional care must be taken to ensure that patients in the “fourth trimester” have these unique needs met and to promote a healthy postpartum experience.

### Electronic supplementary material

Below is the link to the electronic supplementary material.

Supplementary Material 1

## Data Availability

The interview data collected during the current study are available from the corresponding author on reasonable request.

## Abbreviations

HDP: Hypertensive disorders of pregnancy
ACOG: American College of Obstetricians and Gynecologists
