# Supplementary material for: The patient experience of a postpartum readmission for hypertension: a qualitative study
Source: BMC Pregnancy Childbirth. 2024 May 14;24:358. doi: 10.1186/s12884-024-06564-2 (PMC11094995; doi:10.1186/s12884-024-06564-2)
Supplement: Supplementary file 1 — Supplementary Material 1 [file 12884_2024_6564_MOESM1_ESM.docx]

**Questions for semi-structured interview of postpartum patients readmitted with hypertension**

1. Tell me about your delivery experience.
2. Were you diagnosed with a hypertensive disorder, like pre-eclampsia or gestational hypertension, prior to discharge? If yes:
   1. Tell me about that.
   2. What do you understand about that diagnosis?
      1. Connection between pre-eclampsia and future risk of hypertension
   3. Were you started on medications?
   4. Were you anxious/concerned about the diagnosis?
   5. Were you given any instructions about monitoring your blood pressure when you went home?
3. What was your plan for blood pressure monitoring at home?
   1. How often?
   2. Who did it?
   3. When did you talk with your doctor or midwife or nurse about the readings?
   4. Did you have a visit by phone or in person after discharge?
      1. If not, did you miss the appointment or was none scheduled?
   5. Did you understand what to be doing at home for your blood pressure monitoring?
   6. Were there any barriers to monitoring at home?
4. Tell me about how it has been at home?
   1. How are you doing?
   2. How is the baby doing?
   3. Who is helping support you?
5. Tell me about the readmission and what happened?
   1. How do you feel about being readmitted?
   2. Do you think anything could have been done to have prevented the readmission?
   3. Is there anything that could have been done differently in the hospital prior to discharge that would have helped or prevented the readmission?
   4. Is there anything that could have been done when you were at home that could have prevented the readmission?
